# BCRP and P-gp relay overexpression in triple negative basal-like breast cancer cell line: a prospective role in resistance to Olaparib

**DOI:** 10.1038/srep12670

**Published:** 2015-08-03

**Authors:** Robin Dufour, Pierre Daumar, Emmanuelle Mounetou, Corinne Aubel, Fabrice Kwiatkowski, Catherine Abrial, Catherine Vatoux, Frédérique Penault-Llorca, Mahchid Bamdad

**Affiliations:** 1 Clermont Université - Université d’Auvergne - ERTICa – EA 4677 - Institut Universitaire de Technologie, Département Génie Biologique, Ensemble universitaire des Cézeaux, B.P. 86 - 63172 AUBIERE CEDEX, France; 2 Clermont Université - Université d’Auvergne - ERTICa – EA 4677. Centre Jean Perrin, 58 Rue Montalembert, BP 392 - 63011 CLERMONT-FERRAND CEDEX France; 3 Clermont Université - Université d’Auvergne - ERTICa – EA 4677. Faculté de médecine, 28 place Henri Dunant, BP 38 - 63001 Clermont-Ferrand, France; 4 UMR 990 INSERM - Université d’Auvergne, BP 184, 63005 CLERMONT-FERRAND CEDEX, France

## Abstract

The triple negative basal-like (TNBL) breast carcinoma is an aggressive and unfavorable prognosis disease. Inhibitors of poly(ADP-ribose) polymerase such as Olaparib could represent a promising targeted therapy but their sensitivity against Multidrug Resistance proteins (MDR), which causes resistance, is not well defined. Thus, our work focused on the analysis of P-gp and BCRP coexpression in the SUM1315 TNBL human cell line, in correlation with Olaparib intracellular concentration. Western blot analyses showed a clear coexpression of P-gp and BCRP in SUM1315 cells. A low cytotoxic Olaparib treatment clearly led to an increased expression of both BCRP and P-gp in these cells. Indeed, after 1.5 h of treatment, BCRP expression was increased with a 1.8 fold increase rate. Then, P-gp took over from 3 h to 15 h with an average increase rate of 1.8 fold, and finally returned to control value at 24 h. HPLC-UV analyses showed that, in the same treatment conditions, the intracellular Olaparib concentration increased from 1 h to 3 h and remained relatively stable until 24 h. Results suggest that the resistance mechanism induced by Olaparib in TNBL SUM1315 cell line may be overpassed if a cytotoxic and stable intracellular level of the drug can be maintained.

Basal-like (BL) carcinomas represent 10–15% of all invasive breast cancers (BC). These tumors are recognized in clinical practice by their “triple negative” (TN) immunophenotype, *i.e.* estrogen receptor, progesterone receptor and HER2 negative, associated with expression of one or more high molecular weight cytokeratins (5/6,14 or 17) and/or endothelial growth factor receptor (EGFR)^1^. The triple negative basal-like (TNBL) carcinoma is an aggressive and unfavorable prognosis disease. It appears frequently in young women, shows an unusual sarcoma-like pattern of metastasis, and is highly associated with constitutive mutations of the BRCA1 gene^2^.

The management of TNBL carcinomas is not standardized yet. It is based on the use of classical cytotoxic drugs *i.e.* anthracyclines and/or taxanes, although conventional chemotherapy is not always effective in these tumors. Indeed, due to their high proliferative capacity, TNBL tumors first respond to neoadjuvant treatments, but a systematic relapse is then detected, probably caused by resistance^3^. There is an urgent need for more specific therapies for TNBL and BRCA1-mutated tumors^2^. Therapeutic progress relies on a better understanding of underlying molecular alterations that occur in these tumors. BRCA1 and BRCA2 play a major role in DNA repair. As a consequence, their defects lead to genetic instability and resistance to the classical drugs used in BC treatment, *i.e.* anthracyclines and taxanes^4^,^5^.

Thus, new targeted therapies in general and inhibitors of poly(ADP-ribose) polymerase (PARP) in particular could represent a promising therapeutic approach for this type of cancer^6^,^7^,^8^. BRCA1 and BRCA2 genes regulate the repair of damaged DNA *via* the homologous recombination (HR) mechanism, one of the DNA repair pathway^9^,^10^. Therefore, TNBL tumors, which are often BRCA1/2 deficient, are HR-deficient^2^. PARP is a nuclear enzyme involved in another DNA repair pathway called base excision repair (BER)^10^. Through the concept of synthetic lethality, inhibition of PARP in TNBL-BRCA deficient cells causes tumor cells death^11^. Several PARP inhibitors are currently under clinical trial, including Olaparib (AZD 2281; AstraZeneca, London, UK). About ten clinical studies are underway to test Olaparib for breast and ovarian cancer treatments^12^. However, despite the great interest in this type of targeted therapy, several anti-PARPs resistance mechanisms have been identified by *in vitro* and *in vivo* preclinical approaches. The PARP inhibitors action can be altered, either by molecular mechanisms involving the mode of operation of anti-PARPs such as partial HR deficiency, genetic reversion of BRCA mutations or loss of PARP1 expression, or by the Multidrug Resistance transporters (MDR) resistance mechanism^13^,^14^,^15^,^16^,^17^.

The so-called MDR proteins were discovered as membrane transporters inducing chemotherapy resistance in cancer. MDR transporters belong to the “ATP-binding cassette” (ABC) superfamily of proteins, which were highly conserved during evolution. Indeed, these proteins are naturally expressed in all living organisms from prokaryotes to eukaryotes^18^. The two major MDR proteins, Permeability-glycoprotein (P-gp) and Breast Cancer Resistance Protein (BCRP), which belong to the ABCB and ABCG subfamily, respectively, have been showed to be frequently expressed in human cancer. MDR proteins act as xenobiotics efflux pumps able to transport various drugs out of cells. They recognize a large variety of substrates with different structures and properties, including many classical chemotherapeutic agents^19^. Target substrates of these transporters are mainly hydrophobic, but also ionic amphipathic molecules. The MDR system complexity is particularly reflected in the fact that one compound can be the substrate of several MDR transporters. During evolution, the MDR system has formed a cell membrane-localized network that protects the cell from xenobiotics. At present, MDR transporters are considered to be the essential part of an innate cellular defense system, the “chemoimmunity” network, which has a number of reminiscent features of classical immunology^19^.

In this context, the present work focused on the analysis of P-gp and BCRP coexpression in the SUM1315 TNBL human cell line, in correlation with Olaparib intracellular concentration.

## Results

### BCRP and P-gp coexpression in SUM1315 TNBL cell line

**Figure 1:**
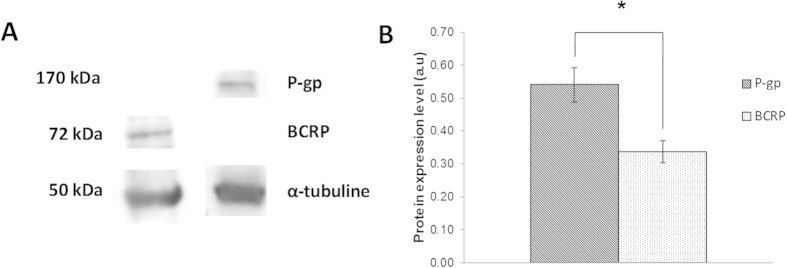
Basal coexpression of BCRP and P-gp in SUM1315 cell line. (**A**) P-gp and BCRP expression were analyzed using Western blot analysis. P-gp and BCRP weighed 170 kDa and 72 kDa, respectively. α-tubulin was used as internal control and presented a molecular weight of about 50 kDa. The full-length blot is presented in Supplementary Fig. S1. (**B**) P-gp and BCRP expression level was quantified using QuantityOne software (Biorad®). Statistical analyses were performed by student’s t test. *indicates a significant difference (p < 0.05) in P-gp and BCRP expression.

Intrinsic expression of BCRP and P-gp was studied in SUM135 cell line, using Western blot analysis. Results showed that the monoclonal anti-P-gp antibody C219 and the anti-BCRP MAB4146 antibody clearly marked two proteins of 170 kDa and 72 kDa, respectively. More, the monoclonal anti-tubulin antibody DM1A marked a tubulin protein with a molecular weight of about 50 kDa, which was used as the internal protein control (Fig. 1A and supplementary Fig. S1). The expression level of BCRP in TNBL cells was 0.34 ± 0.03 a.u. and that of P-gp was 0.54 ± 0.05 a.u. (Fig. 1B).

### SUM1315 cell viability in presence of increasing concentrations of Olaparib

**Figure 2:**
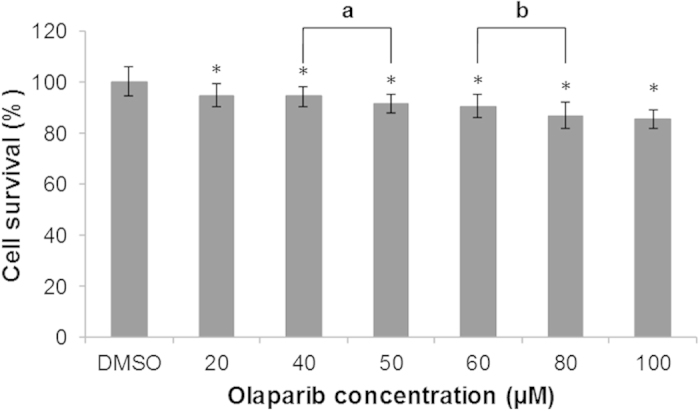
Cell survival rate of SUM1315 cell line in presence of increasing Olaparib concentration. Cell viability was analyzed using the SRB cell viability assay. Statistical analyses were performed by student’s t test. *indicates a significant difference (p < 0.05) in cells survival between 0.1% DMSO control and Olaparib treated cells. “a” and “b” indicate a significant difference (p < 0.05) in cells survival between two concentrations of Olaparib.

The rate of SUM1315 cell viability was analyzed after treatment with increasing Olaparib concentrations (20, 40, 50, 60, 80 and 100 μM) during 24 h, using sulforhodamine B (SRB) viability assay. In parallel, a 0.1% dimethylsulfoxide (DMSO) control without Olaparib was also performed. The results (Fig. 2) showed that the SUM1315 cell viability was 100 ± 5% for 0.1% DMSO control. 0.1% DMSO had no effect on the cell viability. In presence of 20 to 40 μM Olaparib, the cell viability rate decreased slightly but significantly (a mean 94 ± 4%) as compared to control values. For 50 and 60 μM Olaparib, the cell viability rate decreased to a mean of about 90 ± 3%. In presence of higher Olaparib concentrations (80 and 100 μM) the viability cell rate decreased to 85 ± 5%.

### Coexpression kinetic level of BCRP and P-gp in SUM1315 cell line in presence of Olaparib

**Figure 3:**
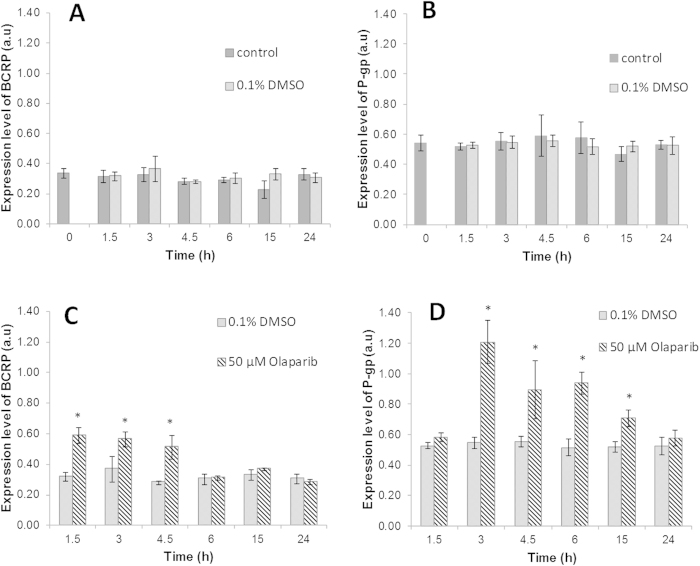
P-gp and BCRP coexpression induction in SUM1315 cell line in the presence of Olaparib. P-gp and BCRP expression levels were analyzed in total protein extract from SUM1315 cells using Western blot analysis. Protein expression was quantified using QuantityOne software (Biorad®). (**A**) Expression level of BCRP in untreated cells and cells treated with 0.1% DMSO. (**B**) Expression level of P-gp in untreated cells and cells treated with 0.1% DMSO. (**C**) Expression level of BCRP in 0.1% DMSO control cells and in cells treated with 50 μM Olaparib. (**D**) Expression level of P-gp in 0.1% DMSO control cells and in cells treated with 50 μM Olaparib. Statistical analyses were performed by student’s t test. *indicate a significant difference (p < 0.05) in BCRP or P-gp expression between control 0.1% DMSO and Olaparib treated cells.

The kinetic of BCRP and P-gp coexpression levels in SUM1315 cell line was then investigated during 24 h, with or without 50 μM Olaparib treatment, using Western blot analysis (Fig. 3). Control and DMSO 0.1% control cells were also performed (Fig. 3A,B). The BCRP expression levels remained stable from 1.5 h to 24 h in the control cells (0.31 ± 0.04 a.u. and 0.33 ± 0.04 a.u., respectively) and in the 0.1% DMSO controls (0.32 ± 0.03 and 0.31 ± 0.03 a.u., respectively). Statistical analysis showed no significant difference between control cells and the 0.1% DMSO control. Therefore, 0.1% DMSO had no effect on the expression level of BCRP in TNBL SUM1315 cell line (Fig. 3A). In parallel, P-gp expression levels in the SUM1315 cell line were measured during 24 h (Fig. 3B,D). As for BCRP, the P-gp expression levels remained stable from 1.5 h to 24 h in control cells (0.52 ± 0.02, 0.53 ± 0.03 a.u., respectively) and in 0.1% DMSO control (0.53 ± 0.02, 0.52 ± 0.06 a.u., respectively). Similarly, statistical analysis showed no significant difference between control cells and 0.1% DMSO control, for P-gp expression during the experiment. Consequently, 0.1% DMSO had no effect on the P-gp expression in SUM1315 cell lines (Fig. 3B).

The kinetic coexpression of BCRP and P-gp in SUM1315 cell cultures was then analyzed after 1.5 h, 3 h, 4.5 h, 6 h, 15 h and 24 h of treatment with 50 μM Olaparib (Fig. 3C,D). After adding 50 μM Olaparib in cell culture, BCRP expression (Fig. 3C) increased significantly from 1.5 h and remained high until 4.5 h (0.59 ± 0.05 a.u. and 0.51 ± 0.08 a.u., respectively). In control 0.1% DMSO control, the BCRP level was 0.32 ± 0.04 a.u. at 1.5 h and 0.28 ± 0.01 a.u. at 4.5 h. Then, the rate of BCRP expression decreased and remained relatively close to controls from 6 h to 24 h (0.31 ± 0.02 a.u. and 0.28 ± 0.02 a.u., respectively). In the same TNBL treated cell culture, P-gp expression level increased significantly from 3 h to 6 h after 50 μM Olaparib treatment (Fig. 3D) (1.21 ± 0.14 a.u. and 0.94 ± 0.07 a.u. against 0.55 ± 0.04 a.u. and 0.52 ± 0.05 a.u. for 0.1% DMSO control, respectively). Then, the P-gp expression level slightly decreased at 15 h (0.71 ± 0.15 a.u.) but still remained significantly higher than the control 0.1% DMSO (0.52 ± 0.03 a.u.). After 24 h of treatment, the level of P-gp expression returned to the control level (0.58 ± 0.05 a.u. for P-gp and 0.52 ± 0.06 a.u. for 0.1% DMSO control).

### Intracellular quantification of Olaparib in SUM1315 cell line

In parallel of MDR proteins expression level studies, a high performance liquid chromatography analytical method with UV detection (HPLC-UV) was developed. This method was used to determine the intracellular quantity of Olaparib in SUM1315 cells after a 50 μM treatment with the drug. The analytical method was used to determine intracellular Olaparib quantities both in cells and in the cell media after different incubation times.

The retention time (t_*R*_) for Olaparib under the chromatographic conditions was 6.4 min. It was checked each day an experiment was performed with a freshly prepared standard solution of the drug in acetonitrile. The UV detection was performed at 218 nm and compared with the standard Olaparib UV spectrum (200–380 nm). Quantitative analyses were performed at 1, 2, 3, 6, 15 and 24 hours. Results are normalized to the highest concentration value and are expressed in percentage. For each incubation time, an untreated control sample was prepared and injected on the chromatographic system.

**Figure 4:**
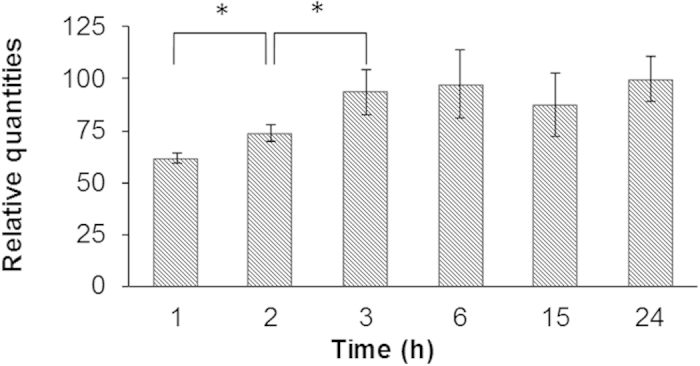
Intracellular Olaparib quantification in SUM1315 cell line over time. For intracellular Olaparib quantification, analytical HPLC experiments were performed. Olaparib was detected at 218 nm and quantified by measuring the area under the curve (AUC) of the corresponding chromatographic peak. Results are expressed in percentage of the highest AUC value.

Results of intracellular Olaparib quantification are shown in Fig. 4. Olaparib was detected at every time points, from 1 to 24 h. The Olaparib level in TNBL cells increased regularly over time from 1 h to 3 h and then remained stable until 24 h. It increased significantly (p < 0.05) from 1 h to 3 h (61.9 ± 2.2% and 93.9 ± 10.8%, respectively). Then, the quantity of Olaparib remained steady until 24 h with no statistically different variation. No degradation product was detected. Also, analysis of the culture media showed that the Olaparib extracellular level remained stable until the latest 24 h time point (Supplementary Fig. S2), and that Olaparib was stable under the incubation conditions as no degradation product was observed.

### SUM1315 cell viability in presence of 50 μM Olaparib over time

**Figure 5:**
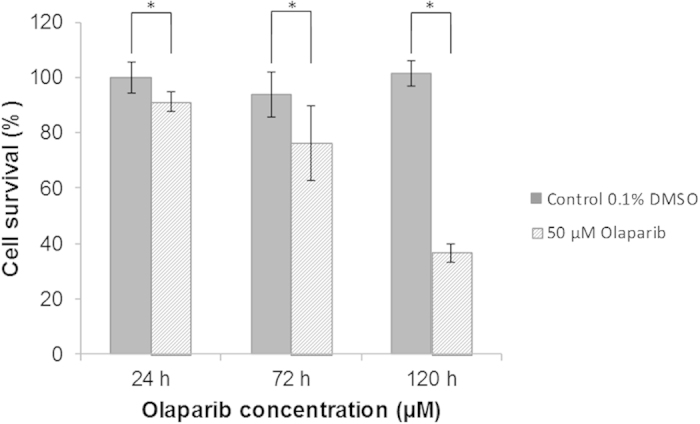
Cell survival rate of SUM1315 cell line in presence of 50 μM Olaparib over time. SUM1315 cells were treated with 50 μM Olaparib and the cell viability was analyzed with the SRB cell viability assay after 24, 72 and 120 h of treatment. Statistical analyses were performed by student’s t test. *indicate a significant difference (p < 0.05) in cells survival between controls and treated cells.

The rate of SUM1315 cell viability was analyzed in presence of 50 μM Olaparib until 120 h, using sulforhodamine B (SRB) viability assay. In parallel, a 0.1% dimethylsulfoxide (DMSO) control without Olaparib was also performed. The results (Fig. 5) showed that the SUM1315 cells viability was 100 ± 5% for the 0.1% DMSO control. In presence of 50 μM Olaparib, the cell viability rate decreased to 90 ± 3%, 76 ± 13% and 36 ± 4% after 24, 72 and 120 h, respectively.

## Discussion

Breast cancer is one of the most diagnosed diseases in the world. Among the subtypes of breast cancer, the TNBL phenotype, which affects mainly young women, is particularly aggressive and presents a poor prognosis. Indeed, the lack of estrogen, progesterone and HER2 receptors expression in these tumor cells makes them insensitive to targeted anti-hormone and/or anti-HER2 therapies^1^. As a consequence, the main treatment remains conventional chemotherapy, with good initial response in some cases, but the tumor is rarely eradicated. Therefore, new targeted therapies represent an urgent need^20^,^21^. PARP inhibitors, a new class of targeted drugs, specifically targeting TNBL tumor cells is being developed and is currently being tested in clinical research^6^,^7^. Based on the synthetic lethality concept, anti-PARPs specifically target TNBL-BRCA deficient tumor cells, thus causing their death^11^. Olaparib (AZD 2281; AstraZeneca, London, UK) is an anti-PARP, which was recently approved by the US Food and Drug Administration and European commission for the treatment of advanced ovarian cancer in patients with BRCA mutations. This drug is also currently in clinical research for various cancers such as solid tumors, breast, prostate, lung, esophagi, gastric, colorectal and brain and central nervous system cancers. In these studies, Olaparib is either used alone or in combination with other anticancer drugs^12^. Regarding breast cancer, several clinical studies are currently underway with Olaparib on triple negative breast cancer^12^. However, despite the great interest in this type of targeted therapy, three mechanisms of resistance to PARP inhibitors have been identified. Namely, secondary BRCA gene mutations, the loss of PARP target protein expression, and the upregulation of MDR efflux transporters^13^,^15^,^17^,^22^.

MDR proteins were identified long ago as a major cause of drug resistance in tumor cells. This defense system causes the efflux of cytotoxic drugs outside the tumor cells^19^. At present, ABCB1/MDR1/P-gp, ABCC1/MRP1, ABCC10/MRP7 and ABCG2/BCRP/MXR are recognized as the major clinically relevant MDR transporters^14^. In this context, the present work focused on the respective implication of P-gp and BCRP, in SUM1315 TNBL cell line model, in response to the PARP inhibitor Olaparib.

The analysis of the expression of P-gp and BCRP in untreated TNBL SUM1315 cell line showed a clear basal coexpression of the two MDR proteins. However, P-gp expression was 1.6 times higher than BCRP. These results suggest that this mechanism of cellular resistance is already present in this TNBL cell line before treatment. Interestingly, the basal coexpression of these two MDR proteins may be partly responsible for the TNBL tumors aggressiveness and poor outcomes reported in clinical practice. Little data exists on the implication of MDR proteins in TN tumors. For instance, it was reported that chemotherapy treatments on TN breast cancers could be individualized and their efficiency rose based on the expression patterns of P-gp obtained from immunohistochemical analyses of patients’ biopsies^23^. Regarding BCRP, it was reported that the IMP3 protein promotes chemoresistance in SUM1315 and MDA-468 TNBL cell lines by regulating this MDR protein expression^24^. Moreover, BCRP expression was also clearly detected in side population of MDA-MB-231 TNBL cell lines^25^.

After basal co-expression of P-gp and BCRP analysis, the impact toxicity of Olaparib was analyzed in SUM1315 cell line in presence of increasing concentrations of drug, ranging from 20 to 100 μM. SRB viability test highlighted few toxicity of Olaparib on these TNBL cell line, with a decrease of about 10% of cell viability at 50 μM and 15% at 100 μM, after a 24 h exposure. The low toxicity concentration of 50 μM Olaparib was then chosen for the next experiments.

To analyze the “resistance response” of TNBL tumor cells against Olaparib in SUM1315 model cell line, the coexpression of P-gp and BCRP was studied in relationship with intracellular concentration of this anti-PARP, during 24 h. The coexpression pattern of BCRP and P-gp over time with a 50 μM Olaparib treatment clearly showed relay overexpression of the two MDR proteins. Indeed, after 1.5 h of treatment, only BCRP expression was clearly increased, with a rate 1.8 fold higher than the control. BCRP expression level remained stable and still high to 4.5 h and then returned to control level at 6 h. P-gp expression level was clearly induced after BCRP, from 3 h to 15 h of treatment, with an average rate of 1.8 fold higher than control. P-gp expression level decreased to the control value after 24 h. These results first showed that Olaparib clearly induced the expression increase of both MDR proteins in this TNBL cell line, which may represent a defense system against this drug. Interestingly, Rucaparib, another PARP inhibitor, was also showed to be transported by both BCRP and P-gp, in polarized monolayers MDCKII human cell line^26^. In our study, the coexpression pattern in SUM1315 cell line of both MDR against Olaparib suggests that BCRP would constitute the first line of defense against this drug. This defense line was strengthened and then relayed by P-gp. All these results suggest that in TNBL tumors, a resistance network settles rapidly against Olaparib. The recognition of Olaparib by P-gp was already reported in *in vitro* models such as in the overexpressing drug-resistant IGROVCDDP cisplatin-resistant ovarian cancer cell line, in the KB-8-5-11 multidrug resistant epidermal carcinoma cell line and in BRCA1-mutated mouse mammary tumors^27^,^28^. In *in vivo* mouse model, it was shown that the resistance to Olaparib could be acquired by the upregulation of Abcb1a/b genes in a TN tumor model^16^.

In parallel of MDR proteins expression studies, the intracellular level of Olaparib in SUM1315 cell line treated in the same experimental conditions was investigated. To that end, a new high performance liquid chromatography method was developed. Results showed that Olaparib rapidly enters the cells. Intracellular Olaparib quantities in cells increased steadily and significantly from 1 h to 3 h (52% increase) and then remained stable at the same level until 24 h. No degradation products were detected in cell samples or in the culture media, which showed that Olaparib was stable under the incubation conditions even after 24 hours. Moreover, the Olaparib concentration in the culture media remained constant at each time point.

Overall, our results show that the presence of Olaparib in the intracellular milieu leads to the significant overexpression of BCRP. That first line of defense settles very rapidly after the beginning of drug exposure and, while dropping back to its basal level, is relayed by the marked overexpression of P-gp. Interestingly, the P-gp overexpression occurs after 3 h, when the intracellular Olaparib reaches its highest level. As mentioned before, this intracellular level remained stable at later time points until 24 h, but the MDR protein expression slowly returned to basal level at 24 h. Thus, in presence of a low toxic Olaparib concentration, a stable level of PARP inhibitor remains into cells despite the early induction of MDR proteins. This intracellular level of Olaparib led to the establishment of a progressive cytotoxicity in these cells, which could explain the return to basal expression of both MDR proteins that was observed. This hypothesis was further supported by the significant decrease in cell viability that was observed after a longer term of 50 μM Olaparib treatment.

In conclusion, a BCRP and P-gp relay overexpression induced by Olaparib in TNBL SUM1315 cell line may be involved in resistance to PARP inhibitor Olaparib. Our results further suggest that this resistance mechanism may be overpassed if a cytotoxic and stable intracellular level of the drug can be maintained. As Olaparib adverse effects are moderate and generally well manageable, this strategy could be adapted to clinical settings and foster the optimization of TNBL Olaparib treatment regimen.

## Methods

### Chemicals and Reagents

All chemicals, unless otherwise noted, were acquired from Sigma-Aldrich (France) and were used as received without further purification. Olaparib was obtained from Sequoia Research Products (Pangbourne, UK). Sulforhodamine B (SRB) was purchased from Sigma-Aldrich (France). All chemicals and solvents used were of HPLC or analytical-reagent grade, and water was purified using Milli-Q gradient A10 (Merck Millipore, UK). All the solvents were filtered through a 0.45 μm Durapore PVDF membrane filter (Merck Millipore, UK) before being used in the chromatographic system. DMSO was of molecular biology grade.

### SUM1315 Cell Culture

SUM1315 cells were obtained from Asterand (Royston, UK) and maintained by weekly serial passage in a 5% CO_2_ atmosphere at 37 °C. Cells were cultured in Ham’s F12 medium (GIBCO^TM^, France) supplemented with 5% fetal bovine serum, 10 mM HEPES buffer, 20 μg/mL gentamicin, 10 ng/mL EGF and 4 μg/mL insulin.

### Preparation of Olaparib solutions and cell culture exposure

Olaparib was dissolved in DMSO as a stock solution at 50 mM. For each experiment, 250 000 cells/ml were first seeded in 5 ml of culture medium and incubated at 37 °C during 24 h for cell adhesion to the flask. The cell cultures were then exposed to 50 μM Olaparib during 1.5 h, 3 h, 4.5 h, 6 h, 15 h and 24 h. For each Olaparib concentration studied, the required volume of Olaparib was removed from the Olaparib stock solution. This was then supplemented with the appropriate volume of DMSO, so that the final DMSO concentration remained always constant *i.e.* 0.1%, in all analyzed cell cultures. In parallel, two controls (i) untreated cells and (ii) cells treated with 0.1% DMSO were carried out.

### Cell Viability Assay

SUM1315 cell viability was measured using the Sulforhodamine B (SRB) colorimetric assay^29^. For this, 5000 cells/well in 200 μL of culture medium were seeded in 96-well microplates and incubated for 24 h at 37 °C and 5% CO_2_, for cells adhesion to the well. In each microplate, a range of controls (untreated cells), controls 0.1% DMSO was performed. In a first series of assays, increasing Olaparib concentrations (20, 40, 50, 60, 80 and 100 μM) were tested and the cell viability was measured after 24 h of incubation. In another series of assays, a fixed 50 μM Olaparib concentration was chosen and the cell viability was measured after various incubation times (24 h, 48 h and 120 h). The final DMSO concentration in each well was always 0.1%. After treatment, cell culture was fixed with trichloroacetic acid at the final concentration of 10% per well. After 1 h incubation at 4 °C, the medium was removed. Cells were washed three times with distilled water. Then, cells were stained by adding 50 μL of 0.4% SRB in 1% acetic acid solution, for 15 min. After three washing with 1% acetic acid, each well was treated with 200 μL of Tris base (10 mM; pH 10.5) and the plate was stirred 30 min for homogenization at room temperature. The absorbance of each well was measured at 540 nm using a microplate reader (Multiskan FC, Thermo Scientific). The viability rate of control cells was determined by the ratio of 0.1% DMSO treated cells/untreated cells. For Olaparib treated cells, viability rate was determined for each concentration by calculating the ratio Olaparib treated cells/0.1% DMSO treated cells.

### MDR expression studies by Western blot analysis

For each experiment, with or without Olaparib treatment, SUM1315 cell cultures pellet were washed with phosphate buffered saline (PBS) (2 × 5 mL), trypsinized (1X trypsin) and re-suspended in culture medium (10 mL) before centrifugation at 4 °C (250 g, 10 min). Cell lysates were prepared from cell pellets using a lysis buffer of 20 mM tris-HCl, 2 mM EDTA, 2 mM EGTA, 6 mM 2-mercaptoethanol (100 μL) supplemented with 1% protease inhibitor (Protease Inhibitor Cocktail P8340, Sigma-Aldrich, France) in conjunction with sonication (5 × 3s) at 4 °C. Protein concentration was determined using a protein assay kit (Coomassie Plus – The Better Bradford Assay^TM^ Kit, Thermo Scientific, France) according to the manufacturer’s protocol. Western blotting was then carried out according to Valton *et al.*, 2013^18^. Briefly, each lysate was diluted in a sample buffer (1.25 M tris-HCl, 20% sodium dodecyl sulfate, 20% glycerol, 2 M dithiothreitol, 0.5% bromophenol blue, pH 6.8) to a protein concentration of 3 μg/μL, and 20 μL of each diluted sample were electrophoresed. P-gp and BCRP were probed using the C219 monoclonal antibody (Calbiochem, Millipore, France) at a 1:500 dilution and the MAB4146 monoclonal antibody (Calbiochem, Millipore, France) at a 1:400 dilution, respectively, and a goat anti-mouse secondary antibody conjugated to horseradish peroxidase (HRP) (Promega, France) at a 1:10000 dilution. The α-tubulin loading control was probed using the DM1A mouse monoclonal antibody at a 1:100000 dilution and the same HRP conjugated secondary antibody. Immune complexes were visualized by chemiluminescence using the ECL2-enhanced chemiluminescence detection system (Pierce, Thermo Scientific, France) according to the manufacturer’s specifications. Densitometric analyses were performed using Quantity-One software (version 4.6.6, Bio-Rad, France). P-gp or BCRP expression was determined by calculating the ratio of the density of the corresponding band over the density of the α-tubulin band. This ratio was expressed in arbitrary unit (a.u.).

### High performance liquid chromatography for intracellular Olaparib quantification

For intracellular Olaparib quantification, analytical HPLC experiments were performed on a HP 1100 series LC system equipped with an online degasser, a quaternary pump, a column oven, a photodiode array detector and an autosampler. Solvents A (H_2_O with 0.1% v/v TFA) and B (acetonitrile with 0.1% v/v TFA) used for the preparation of the mobile phase were filtered before use. The temperature of the column oven was kept at 25 °C. A gradient elution was applied at a flow rate of 0.8 mL/min through a reversed-phase Nova-Pak® 4 μm C18 column (150 × 3.9 mm; Waters Corporation, Milford, MA, USA) with an additional Nova-Pak® guard column. The following gradient was employed: 0–11 min, 5–80% B; 11–13 min, 80% B; 13–15 min, 80–5% B; 15–19 min, 20%. 20 μL were injected into the HPLC system. The photodiode array detection was carried out from 200 to 380 nm. Olaparib was detected at 218 nm and quantified by measuring the area under the curve (AUC) of the corresponding chromatographic peak at a retention time of 6.4 min. Results are expressed in percentage of the highest AUC value and the purity of the peak was assessed by comparing the UV spectrum (200–380 nm) with the one obtained with a 50 μM standard solution of Olaparib in acetonitrile. HP Chemstation software (version B.04.03-SP1, Agilent Technologies, France) was used for acquisition of chromatograms and integration data.

SUM1315 cell culture (250 000 cells/mL) was treated with 50 μM Olaparib during 1 h, 2 h, 3 h, 6 h, 15 h and 24 h). After each incubation time, the culture medium was removed and stored at 4 °C for analysis. Cells were subsequently washed with ice-cold PBS (2 × 2 mL) and then lysed by scraping in acetonitrile (2 mL) in conjunction with sonication (5 × 3s). After centrifugation of the samples at 4 °C (350 g, 10 min), the supernatant was filtered through a 0.45 μm PVDF membrane filter and pipetted into a 1 mL autosampler vial for injection.

For each experiment, the concentration of Olaparib in the cell culture medium was also determined. The culture medium was diluted (1:2) with acetonitrile and the mixture was subsequently stirred and centrifuged at 4 °C (350 g, 10 min). The supernatant was filtered through a 0.45 μm PVDF membrane filter and pipetted into a 1 mL autosampler vial for injection.

### Data analysis

All experiments were performed at least in triplicate. Results are presented as means +/**−** standard deviation. Statistical analyses were performed with Microsoft Excel using a two-sided Student’s *t* test. p < 0.05 was considered as significant.

## Additional Information

**How to cite this article**: Dufour, R. *et al.* BCRP and P-gp relay overexpression in triple negative basal-like breast cancer cell line: a prospective role in resistance to Olaparib. *Sci. Rep.* **5**, 12670; doi: 10.1038/srep12670 (2015).

## Supplementary Material

Supplementary Information
