# Supplementary material for: BCRP and P-gp relay overexpression in triple negative basal-like breast cancer cell line: a prospective role in resistance to Olaparib
Source: Sci Rep. 2015 Aug 3;5:12670. doi: 10.1038/srep12670 (PMC4522660; doi:10.1038/srep12670)
Supplement: Supplementary Information [file srep12670-s1.pdf]

Supplementary information:

Title of the manuscript:

BCRP and P-gp relay overexpression in triple negative basal-like breast cancer cell line: a prospective role in resistance to Olaparib.

Authors:

Robin Dufour, Pierre Daumar, Emmanuelle Mounetou, Corinne Aubel, Fabrice Kwiatkowski, Catherine Abrial, Catherine Vatoux, Frédérique Penault-Llorca & Mahchid Bamdad.

Supplementary figure S1: full length blot

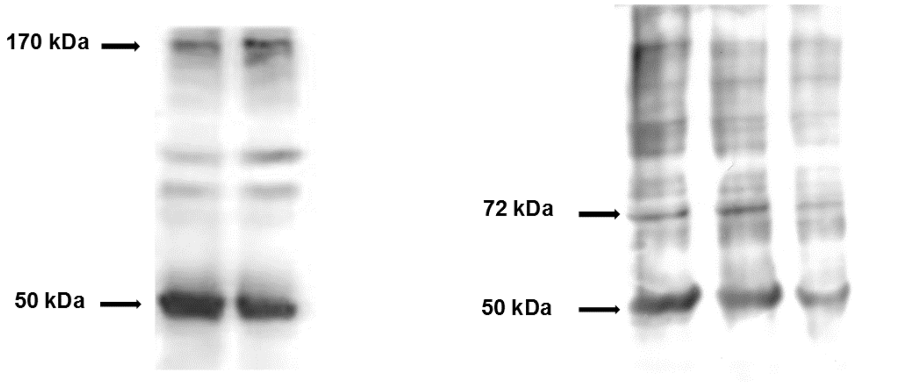

Expression of P-gp in SUM1315 cell line **Expression of BCRP in SUM1315 cell line**

Supplementary figure S2: extracellular Olaparib quantification.

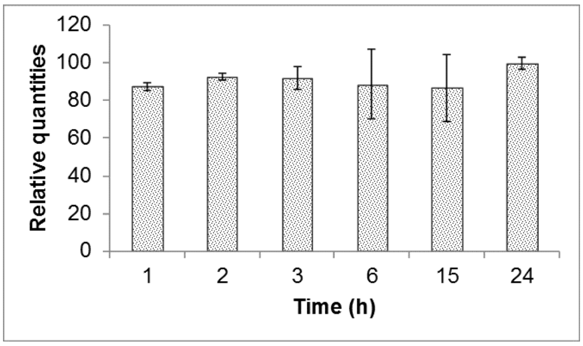

Extracellular Olaparib quantification in SUM1315 cell culture media.
For extracellular Olaparib quantification, analytical HPLC experiments were performed. Olaparib was detected at 218 nm and quantified by measuring the area under the curve (AUC) of the corresponding chromatographic peak. Results are expressed in percentage of the highest AUC value.
